# Distributions of Virus-Like Particles and Prokaryotes within Microenvironments

**DOI:** 10.1371/journal.pone.0146984

**Published:** 2016-01-19

**Authors:** Lisa M. Dann, James S. Paterson, Kelly Newton, Rod Oliver, James G. Mitchell

**Affiliations:** 1 School of Biological Sciences at Flinders University, Adelaide, South Australia; 2 Land and Water Research Division at the Commonwealth Scientific and Industrial Research Organisation (CSIRO), Adelaide, South Australia; University of Aveiro, PORTUGAL

## Abstract

Microbial interactions are important for ecosystem function, but occur at the microscale and so are difficult to observe. Previous studies in marine systems have shown significant shifts in microbial community abundance and composition over scales of micrometres to centimetres. This study investigates the microscale abundance distributions of virus-like particles (VLPs) and prokaryotes in the lower reaches of a river to determine the extent to which microscale microbial patchiness exists in freshwater systems. Here we report local hotspots surrounded by gradients that reach a maximum 80 and 107 fold change in abundance over 0.9 cm for prokaryotic and VLP subpopulations. Changes in prokaryotic and VLP hotspots were tightly coupled. There were no gradients at tens of centimetres across the boundary layers, which is consistent with strong mixing and turbulence-driven aggregation found in river systems. Quantification of the patchiness shows a marked asymmetry with patches 10 times greater than background common, but depletions being rare or absent in most samples. This consistent asymmetry suggests that coldspots depleted by grazing and lysis are rapidly mixed to background concentrations, while the prevalence of hotspots indicates persistence against disruption. The hotspot to coldspot relative abundance may be useful for understanding microbial river dynamics. The patchiness indicates that the mean- field approach of bulk phase sampling misses the microbially relevant community variation and may underestimate the concentrations of these important microbial groups.

## Introduction

Heterotrophic prokaryotes are crucial to freshwater systems as they cycle important nutrients, such as nitrogen, carbon and phosphorus [1, 2]. Similarly, viruses are likely key players in freshwater ecosystems as they affect primary production, nutrient and dissolved organic matter release, genetic exchange between microorganisms and control bacterial community composition and abundance via viral-mediated lysis [3–7]. The distributions of these microbial communities have often been considered homogeneous, leading to mean-field approach sampling whereby bulk phase millilitre to litre samples were considered representative of microbial microscale processes [2]. However, previous studies on patch dynamics within aquatic and terrestrial ecosystems confirm heterogeneous spatial distributions are common in most organisms. For instance, biodiversity ‘hotspots’ are observed in flora and terrestrial vertebrate species [8, 9], with 20% of all plant diversity found in 18 hotspots that accounted for only 0.5% of the Earth’s land area [10, 11]. In addition, for marine environments, it is well accepted that microbial abundance and activity differ by orders of magnitude over micrometres to centimetres [1, 2, 12–16], with previous studies showing 45 to 2584 fold cm^-1^ variation in bacterial and viral abundance [17], and therefore indicating deficiencies within the mean-field sampling approach.

As microbial communities interact at micrometre to millimetres scales, to understand these microscale processes and dynamics, it is important to analyse their distributions at such scales. Microscale sampling is an important supplement when precise abundance estimates are necessary, the local interactions may reveal processes not observed by bulk sampling or for localizing particular populations. Determining the microscale abundance distribution is important as it indicates the microbial structure present at the scale of microbial food webs. Determining the microscale abundance distribution is important as it indicates the microbial structure present at the scale of microbial food webs. However, in freshwater systems, studies of microbial distributions often still adopt the mean-field approach, therefore excluding direct examination of important ecological associations between microbial communities [18–22].

Associations between microbial populations are often observed within microscale distributions in the form of heterogeneous “hotspots” [1, 10–13, 19, 23]. Hotspots are areas of elevated bacterial or viral abundance, which can result from bacterial accumulation around high nutrient concentration areas via chemotaxis, aggregation or disintegration of particles, small scale water mixing and the occurrence of grazing in adjacent areas or interactions with suspended particles [15, 24–26].

Within fluvial systems suspended particulate inorganic and organic matter are often found in high concentrations [27]. This particulate organic matter (POM) can comprise a range of different materials, such as biofilms, soils, riparian vegetation or autochthonous algae, and is an important source of energy as bacterial communities contribute to the decomposition and remineralisation of this POM [28–31]. Previous studies have found higher heterogeneity, and often abundances, within particle-associated, rather than free living communities, with particle-associated bacteria contributing to approximately 30.34% of total bacterial abundance [32–34]. Particle-associated bacterial communities, such as those found on river snow particles, are important to system function as they contribute to a significant amount of production and activity [33, 35–38].

The presence of these particles also impacts viral communities, with viral abundance on suspended particulate matter ranging from 10^5^ to 10^11^ particles ml^-1^ or between 0.4% and 35% of total viral abundance [33, 34]. Viral attachment to particles and/or the presence of particulate matter can result in different ecological consequences; for instance, a loss of infectivity as a result of viral adsorption to solid particles, which causes a reduction in viral infection and lysis and a subsequent increase in free-living prokaryotic growth [29]. In addition, prolonged survival or increased phage production and transduction can result from viral particle attachment as high viral abundances on riverine particles may represent microenvironments of heightened viral infection and lysis of bacterial communities [29, 33, 39–42].

Lysis events will produce intense local concentrations of viruses, which may spread as local epidemics. This should produce areas of depleted bacterial concentration. However, this is not observed in turbulent environments, presumably because shear caused by turbulence easily disperses the immotile, non-aggregating microbes. Hotspot formation and maintenance, whether viral or bacterial, is a balance of aggregation and dispersion [43–45]. Dispersion at the microscale in turbulent systems such as rivers is driven by Kolmogorov eddies. These are the smallest possible eddies for a given fluid viscosity [46]. For rivers, where the viscosity is close to that of pure water, the Kolmogorov eddy length is approximately 1–10 mm [47, 48]. When eddies and heterogeneities are the same size, mixing is the most efficient homogenizing signals. This is relevant for this paper because our sampling interval is right at the scale where there should be the most homogeneity [44].

While the high shear of Kolmogorov eddies efficiently erase gradients of microbes and nutrient signals [49–51], many eddies are much larger, which can create conditions for clustering [51]. This is because low shear environments are characterised by eddies with long lifetimes, which allow microscale nutrient patch formation and consequently chemotactic swarming of bacteria. The lifetimes of these Kolmogorov eddies differ depending on turbulence, with freshwater environments having eddy lifetimes of approximately 1,000 seconds and lengths of greater than 3 cm [47, 51]. Shear then helps determine where hotspots and coldspots can form, their size and their lifetime [22, 47, 51, 52].

Previously, hotspot discrimination has remained qualitative, identifying abundance regions ‘elevated’ above background. However, a quantitative method developed by Dann *et al*. [17] discriminated hotspot, coldspot and background via rank abundance graphs, separating sample values based on their slope and line of best fit. Hotspots were shown to have steep slopes and follow a power law best fit, coldspots and background values follow linear best fits, while coldspots had steeper slopes than background values [17]. The aim of this study was to test the hypothesis thatmicroscale VLP and prokaryote abundance variations occur in rivers. To test this, the microscale distributions of prokaryote and VLP subpopulations within the Murray River were analysed at millimetre resolution.

## Materials and Methods

### Sample Collection

Freshwater samples were collected from the Murray River at Murray Bridge, South Australia (-35°12, 139°28). Sampling occurred on June 14^th^, 2012 at 11 am. Daily summary data showed a water level of 0.45 metres, and electrical conductivity (EC) of 325 uS/cm (Long Island Site ID: A4261162) [53]. Water flow rates ranged from 0.01 m/s to 0.09 m/s. The flow rates were determined via velocity profiles using a Flo-Mate (Model 2000) current and flowmeter. Velocity profiles were taken via fixed time averaging with 60 second intervals. Specific permission to access the sampling site was not required. The field study did not involve endangered or protected species.

Triplicate samples were collected at the sediment-water and air-water interface from three locations each separated by 10 metres. Samples were collected using a two-dimensional sampler trialled previously [17]. The sampler, a 3 x 12-well microplate with a glass cover collected three vertical profiles, each containing 12 sample points, ranging from 1.4 cm to 11.3 cm from the sediment- and air-water interface with a sampling resolution of 0.9 cm.

Once collected, samples were transferred into 2 ml cryovials containing 4 μl of glutaraldehyde (0.5% final concentration) and stored in the dark at 4°C for 15 minutes. Samples were then quick frozen in liquid nitrogen and stored at -80°C until analysis [54, 55]. Flow cytometric analysis was performed within three weeks to avoid sample deterioration [54].

#### Flow cytometry

For prokaryote and VLP enumeration, samples were thawed and diluted 1:100 with Tris-EDTA buffer (pH 8.0, 0.2 μm filtered, 10 mM Tris, 1 mM EDTA) and stained with SYBR Green I (1:500 final dilution commercial stock; Molecular Probes), a nucleic acid-specific dye [56, 57]. Samples were then incubated in the dark at 80°C for 10 minutes to optimise VLP counts [17, 25, 54, 58, 59]. Each sample was measured in triplicate to check the precision of the method.

Flow cytometry was performed on a FACSCanto II flow cytometer (BD) using a phosphate-buffered saline (PBS) solution as sheath fluid. Forward-angle light scatter (FSC), right-angle light scatter (SSC) and green fluorescence (SYBR I) were collected for each sample. Each sample was run at a low flow rate setting to obtain less than 1000 events per second. Reference fluorescent yellow beads (1 μm diameter, Molecular Probes) were added to each sample as an internal size and concentration standard with flow cytometer settings normalised to fluorescence and bead concentration [25, 59]. Epifluorescent microscopy was used to ensure bead reliability by confirming a final concentration of approximately 10^5^ beads ml^-1^ per sample [57, 59–61]. Flow cytometric data was exported as FCS 3.0 files and prokaryotic and VLP subpopulation enumeration was performed using FlowJo (Tree Star, Inc.) [61]. VLP and prokaryotic subpopulations were discriminated via peaks in monoparametric histograms of SYBR green fluorescence and saturated regions in biparametric cytograms of SYBR green fluorescence and side-scatter [17, 54, 56, 58, 62].

### Microscale Distribution Analysis

The microscale distributions of prokaryotic and VLP subpopulations at the air- and sediment-water interface were determined via flow cytometric abundance counts in each sample well of the vertical profiles collected. From this, two-dimensional contour plots were constructed using Surfer 10 (Golden Software, Inc.). Background values reported are the median values in the dataset rather than the mean, as inclusion or exclusion of the hotspot values did not overly affect the median values [63].

For this study, hotspot and background values were determined via rank abundance graphs [17]. Briefly, background values were those that fitted a linear trend and hence were indistinguishable from a random distribution, whilst the hotspot values exceeded this linear fit, exhibiting a steep power law trend, indicating their non-random nature [17].

### Subpopulation Correlations

Pearson’s correlation coefficients were performed for each vertical profile collected at the air- and sediment-water interface and the α of 0.05 was reduced by sequential Bonferroni [64]. All possible subpopulation correlations were considered in order to identify potential relationships between the VLP and prokaryotic subpopulations. Two-sample t-tests were employed to identify possible abundance differences between the air- and sediment-water interface.

The VLP to prokaryote ratio (VPR) was used as a potential indicator of VLP and prokaryote interactions. Previous studies used the patchine to investigate the biological dynamics of systems [57]. Higher VPRs are typically found in more productive and nutrient-rich ecosystems with the suggestion that these conditions favour maximum prokaryotic growth and productivity [7, 65, 66].

### Spatial Autocorrelation Analysis

Spatial autocorrelation analysis was performed to determine the spatial dependence within prokaryote and VLP communities. Originally used by the National Institute of Justice for identifying non-random spatial patterns in crime occurrences [67] spatial autocorrelation analysis can also be applied to organism distribution and has been used previously to look at the level of spatial complexity within phytoplankton communities [68]; the distribution of cellular nucleic acid signals within floating riverine aggregates [29], the frequency of particular allozymes in snail populations [69]; and the distribution of particular genes in Australian rat species [70]. This study used two common spatial autocorrelation analysis statistics; Moran’s I and Geary’s C, to assess the spatial complexity of the indigenous prokaryote and VLP communities [69, 71, 72]. Moran’s I and Geary’s C identify whether two-dimensional and multi-directional correlations are present between proximate sample points that have similar values [69, 71, 72]. Analysing the spatial autocorrelation in VLP and prokaryotic microscale distributions via Moran’s I and Geary’s C builds on previous work by Dann *et al*. [17].

#### Moran’s I

Moran’s I spatial autocorrelation statistics test (CrimeStat 3.3, Ned Levine software) was used to identify the degree of spatial dependence present in the VLP and prokaryotic subpopulations. The equation for Moran’s I is:

$$I=\frac{N\sum i\sum jW_{ij}\left(X_{i}-\bar{X}\right)\left(X_{j}-\bar{X}\right)}{\left(\sum i\sum jW_{ij}\right)\sum i{\left(X_{i}-\bar{X}\right)}^{2}}$$ Eq. 1

Where *N* refers to the sample number, *X*_*i*_ and *X*_*j*_ are the variable values at specific locations, i and j (where i ≠ j), $\bar{X}$ is the mean of the variable and *W*_*ij*_ is the weight applied to the i and j comparison. A weighted Moran’s I test was chosen as this applies a weight value of 1 and 0 to adjacent and non-adjacent sample points respectively.

Moran’s I [72] is a global statistical test used to identify spatial dependence within a dataset. It is multi-directional as it can use vertical, horizontal and diagonal directional analysis for correlation calculations. Moran’s I has a value range from +1 to -1, with +1 indicating perfect clustering where high/low values are proximate, -1 indicating perfect dispersion where high/low values are found far apart and zero being indicative of a random distribution. For Moran’s I, the critical cut-off values are often established through a collation of previous literature using the same or similar scales or values. This information is lacking for prokaryotes and viruses and the critical cut-off values for small-scale spatial studies are unknown.

#### Moran Correlograms

Moran correlograms were created using CrimeStat 3.3 whereby output values from Moran’s I statistic were applied to two pairs of sample values that were separated by a chosen lag distance, in this instance 0.9 cm, the distance between each well in the sampler used.

Significance was determined by the standard error obtained from Moran’s I and the degree of spatial autocorrelation was determined via the output values given for each lag distance in the correlograms. From this, the level of spatial dependence could be identified at each sample distance as opposed to the whole sampling area, as was achieved in the Moran’s I statistic.

#### Geary’s C

Geary’s C spatial autocorrelation test [71] within CrimeStat 3.3 was used to identify deterministic patterning of extreme values and non-spatially related chance phenomena within the dataset. The equation for Geary’s C is:

$$C=\frac{\left(N-1\right)\left[\sum i\sum jW_{ij}{\left(X_{i}-X_{j}\right)}^{2}\right]}{2\left(\sum i\sum jW_{ij}\right)\sum i{\left(X_{i}-\bar{X}\right)}^{2}}$$ Eq. 2

All terms are the same as in Eq 1.

Geary’s C is more sensitive to local clustering and can be used in conjunction with Moran’s I. The Geary’s C statistical test is similar to Moran’s I, however spatial dependence is calculated via the deviation in intensity of each sample value’s location compared to one another, whereas Moran’s I calculates spatial dependence via the cross-product of the deviations from the mean within the sample values.

Geary’s C has a value range from 0 to approximately 2, with no definitive upper limit [46]. Spatial independence is indicated by a value of 1 whilst positive spatial autocorrelation is indicated by values < 1 and negative spatial autocorrelation is indicated by values > 1. Thus, Geary’s C is inversely related to Moran’s I [73].

## Results

### Prokaryotic and VLP abundance

**Fig 1:**
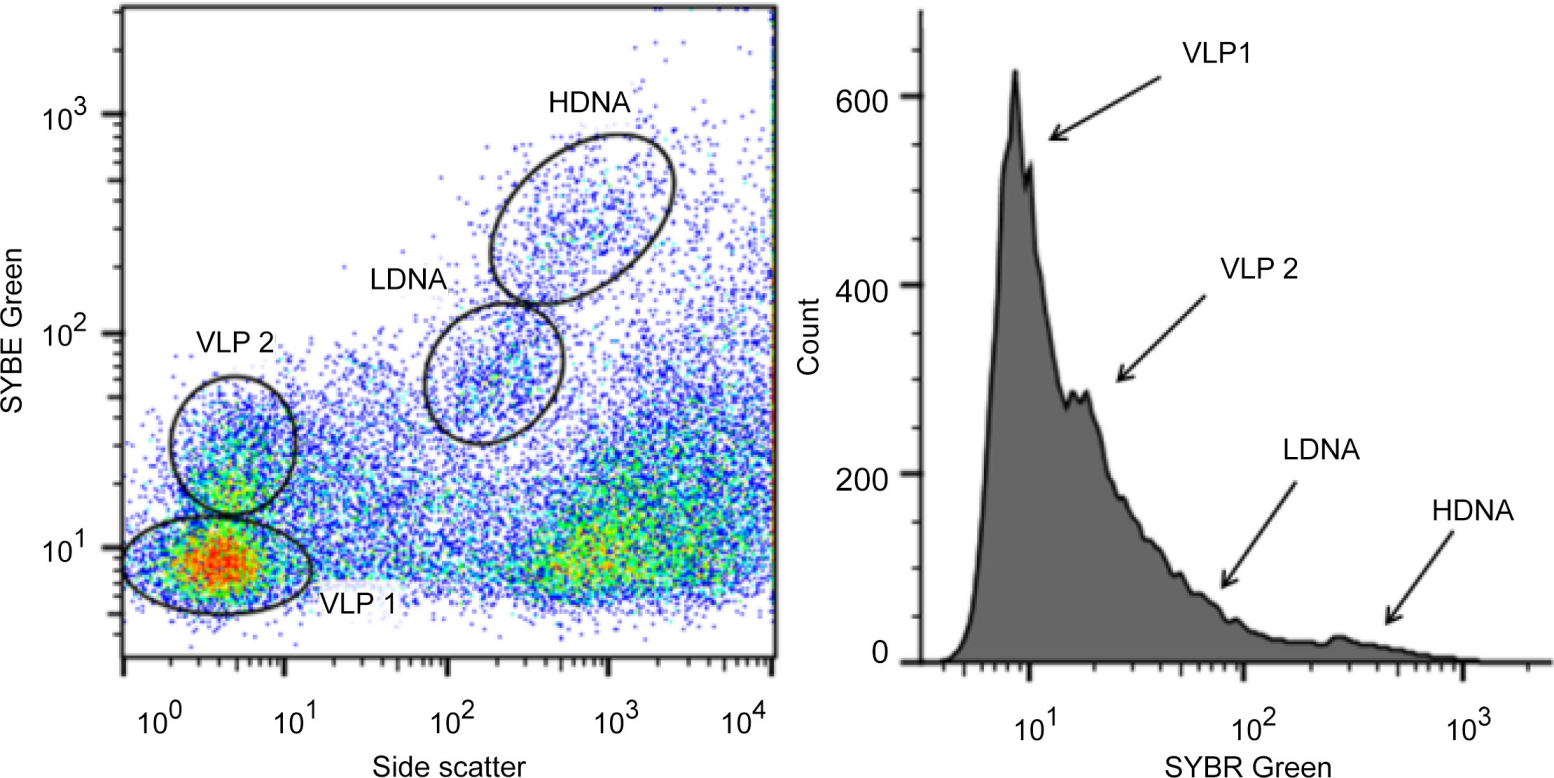
Representative flow cytometric cytogram and histogram. (A) Cytogram of SYBR green fluorescence versus side scatter and (B) histogram of SYBR green fluorescence showing two VLP (VLP 1 and VLP 2) and two prokaryotic (LDNA and HDNA) subpopulations.

Flow cytometric analysis revealed two prokaryotic subpopulations, referred to as a low- and high-density nucleic acid prokaryote population (LDNA and HDNA), and two virus-like particles (VLP 1 and VLP 2) subpopulations (Fig 1). From all the samples collected, the mean VLP 1 abundance ranged from 5.4 to 6.7 x 10^7^ particles ml^-1^ (95%CI = 7.2 x 10^6^ particles ml^-1^, n = 648) whilst mean VLP 2 abundance ranged from 1.7 to 2.1 x 10^7^ particles ml^-1^ (95%CI = 2.3 x 10^6^ particles ml^-1^, n = 648) (S1 Table). The mean prokaryotic abundances were lower than the VLP mean abundances, with the mean abundance of the LDNA subpopulation ranging from 1.3 to 1.6 x 10^7^ cells ml^-1^ (95%CI = 1.3 x 10^6^ cells ml^-1^, n = 648) and the HDNA subpopulation exhibiting mean abundances that ranged from 0.9 to 1.5 x 10^7^ cells ml^-1^ (95%CI = 1.9 x 10^6^ cells ml^-1^, n = 648) (S2 Table). This resulted in a VPR ranging from 0.4 to 3.4 (S3 Table). In all samples, the VLP 1 abundance was higher than VLP 2 and the HDNA abundance was equal to or less than the LDNA abundance (S1 and S2 Tables). There was no significant difference in VLP and prokaryotic mean abundances between the air-water and sediment-water interface (p = 0.39).

### Hotspots

**Fig 2:**
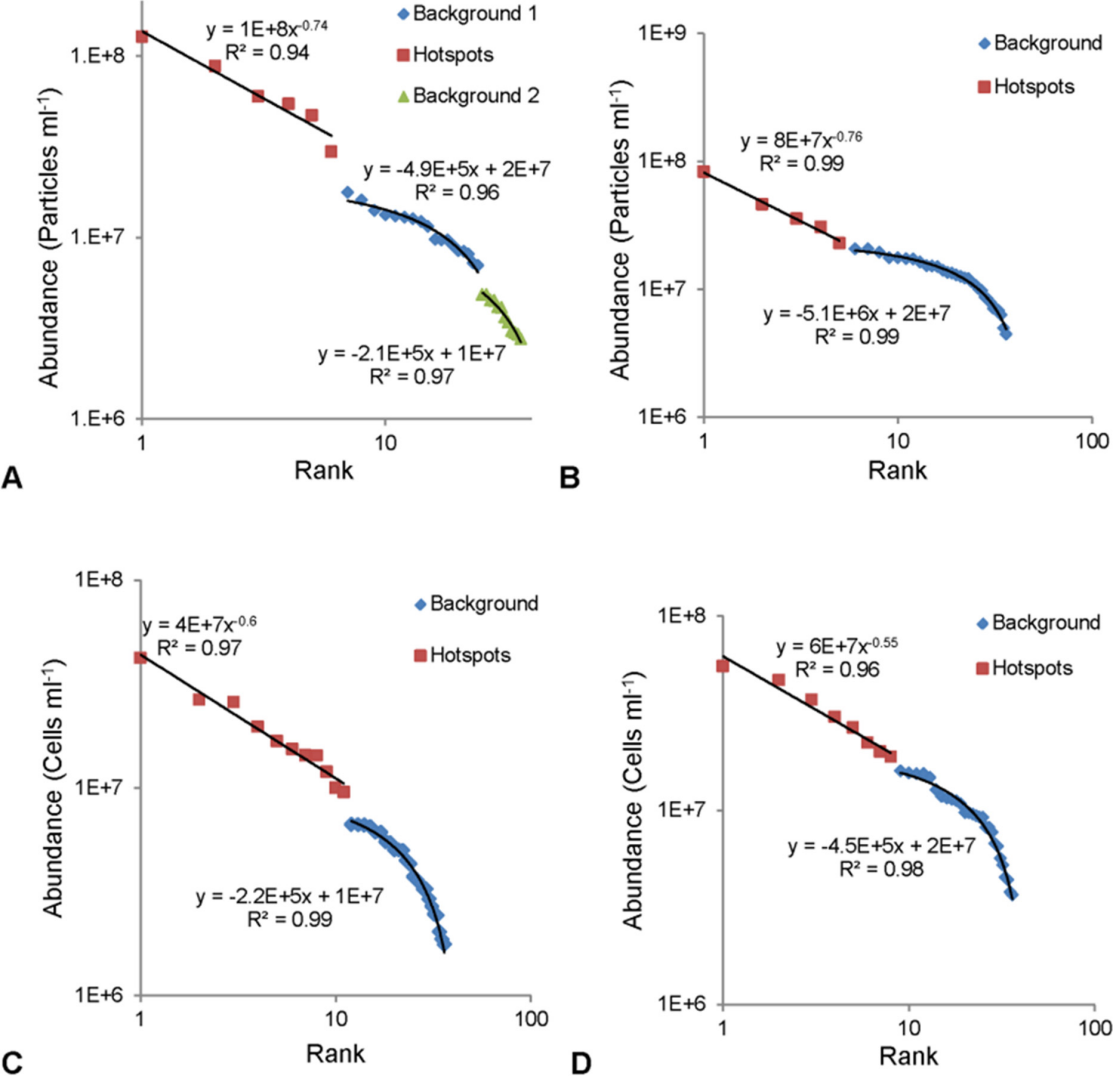
Representative rank abundance graphs for hotspot determination. **Background values followed a linear trend, whilst hotspots exhibited a power law trend.** (A) VLP 2 at air-water interface. (B) VLP 1 at air-water interface. (C) HDNA at sediment-water interface. (D) LDNA at air-water interface.

Rank abundance graphs of the vertical profiles revealed hotspots within all the prokaryotic and VLP subpopulations at the air- and sediment-water interface. In 19 of 24 samples the background values followed a single linear trend whilst the remainder followed two or three linear trends. Background values followed a linear trend with slopes ranging from -2 x 10^5^ and -5 x 10^6^, whilst the hotspot values followed a power law trend with exponents ranging from -0.26 to -1.43 (Fig 2A–2D).

**Fig 3:**
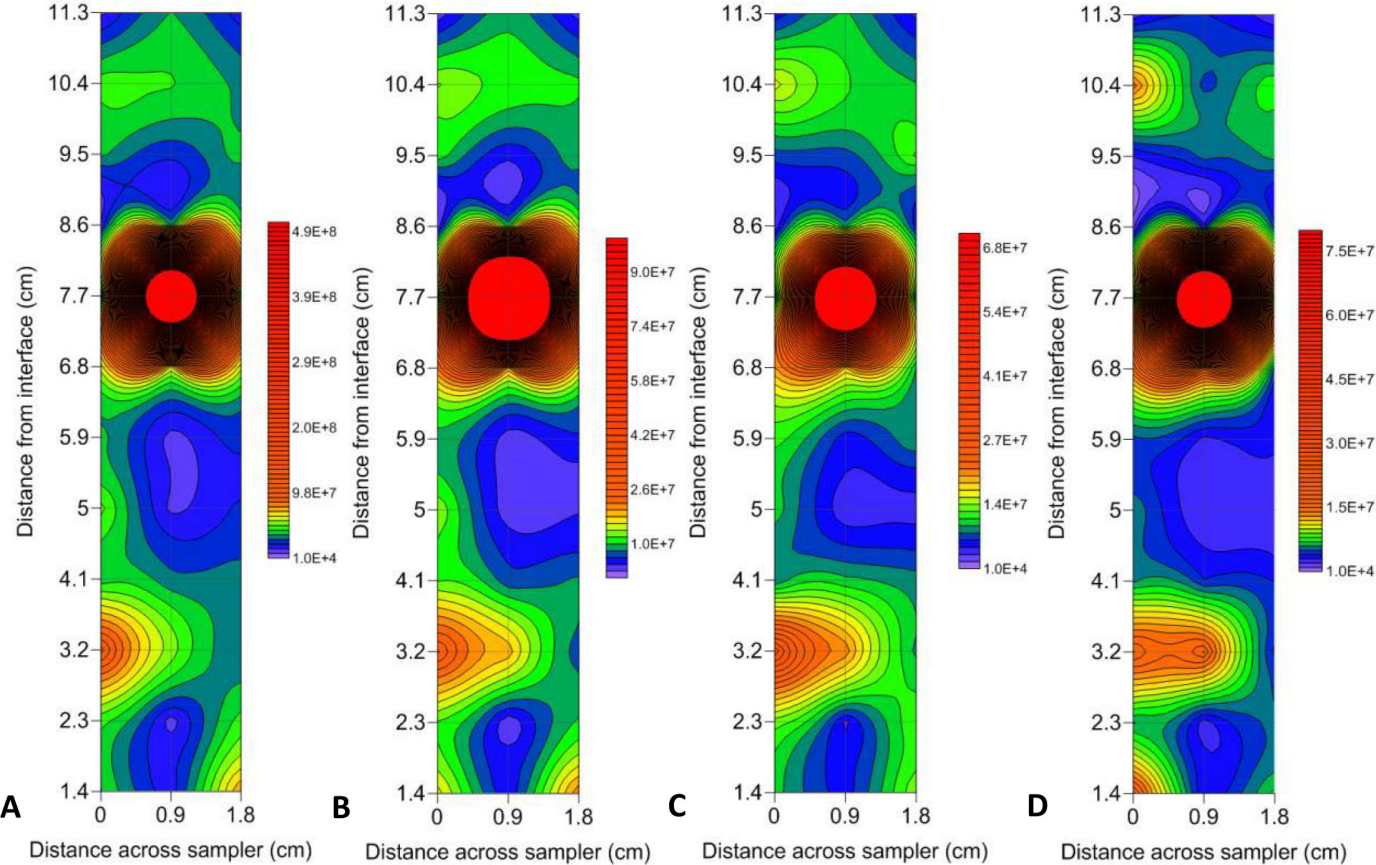
Contour plots showing hotspots in prokaryotic and VLP subpopulations at the sediment-water interface. (A) VLP 1 (B) VLP 2 (C) LDNA (D) HDNA. Abundance levels indicated via a colour intensity scale in units of cells/particles ml^-1^. Faint gridlines indicate sample intervals. A minimum contour interval value of 10000 was chosen as this was larger than the maximum flow cytometer machine error observed in blank control samples. Solid red areas indicate abundance points higher than the maximum contour level selected.

In all instances, the maximum abundance hotspots were present at the sediment-water interface, and when analysing the total 1.8 cm x 11.3 cm sampling area, VLP 1 had a maximum hotspot of 8.5 x 10^8^ particles ml^-1^ with a background of 3.6 x 10^7^ particles ml^-1^, resulting in a 24 fold increase in heterogeneity (Fig 3A). Whereas VLP 2 had a lower maximum hotspot value of 2.6 x 10^8^ particles ml^-1^ but over a background of 1 x 10^7^ particles ml^-1^ resulting in a 26 fold change in heterogeneity, which was higher than VLP 1 (Fig 3B).

As the prokaryotic abundances were lower than the VLP abundances, the maximum abundance hotspots found in LDNA and HDNA were lower than VLP 1 and VLP 2. LDNA had a maximum abundance hotspot of 1.3 x 10^8^ cells ml^-1^ over a background of 9 x 10^6^ cells ml^-1^, resulting in a 15 fold change in heterogeneity over the 1.8 x 11.3 cm sampling area (Fig 3C). HDNA had similar values, with a maximum abundance hotspot of 1.4 x 10^8^ cells ml^-1^ but over a background of 4.6 x 10^6^ cells ml^-1^ resulting in a 31 fold difference in heterogeneity which was higher than LDNA and was the largest change in heterogeneity in all of the subpopulations (Fig 3D).

However, these maximum abundance hotspot values were not the cause for the largest changes in heterogeneity. The largest fold changes were from one sample point to the next, over a distance of 0.9 cm, rather than across the entire sampling area. This was due to the occurrence of single point hotspots adjacent to low abundance values. VLP 1 showed a maximum 74 fold change in heterogeneity over 0.9 cm, whilst VLP 2 had the highest change in heterogeneity showing a maximum 107 fold change over 0.9 cm (Fig 3A and 3B). For the prokaryotic subpopulations, LDNA had a maximum 41.5 fold change over 0.9 cm, whilst HDNA showed a maximum 80.5 fold change over 0.9 cm (Fig 3C and 3D).

### Subpopulation correlations

**Fig 4:**
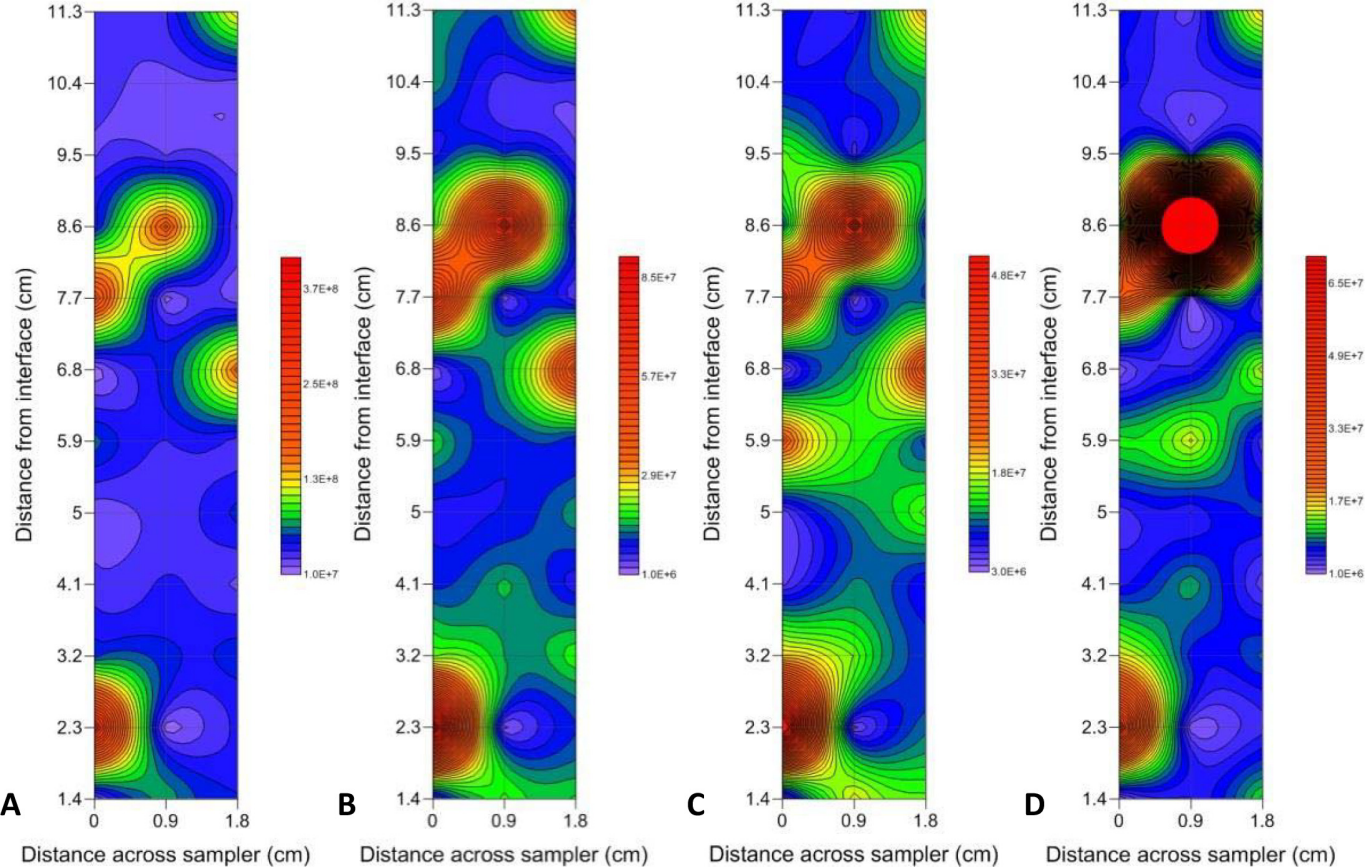
Representative two-dimensional contour plots showing the presence vs. absence of hotspots in prokaryotic and VLP abundance. (A) VLP 1, (B) VLP 2, (C) LDNA and (D) HDNA at the air-water interface. Abundance levels indicated via a colour intensity scale in units of cells/particles ml^-1^. Faint gridlines indicate sample intervals. A minimum contour interval value of 10000 was chosen as this was larger than the maximum flow cytometer machine error observed in blank control samples. Solid red areas indicate abundance points higher than the maximum contour level selected.

Two-dimensional contour plots revealed that the prokaryotic and VLP subpopulations were correlated overall, but that there were locational differences caused by the presence or absence of hotspots in prokaryotic and VLP abundance (Fig 4).

**Fig 5:**
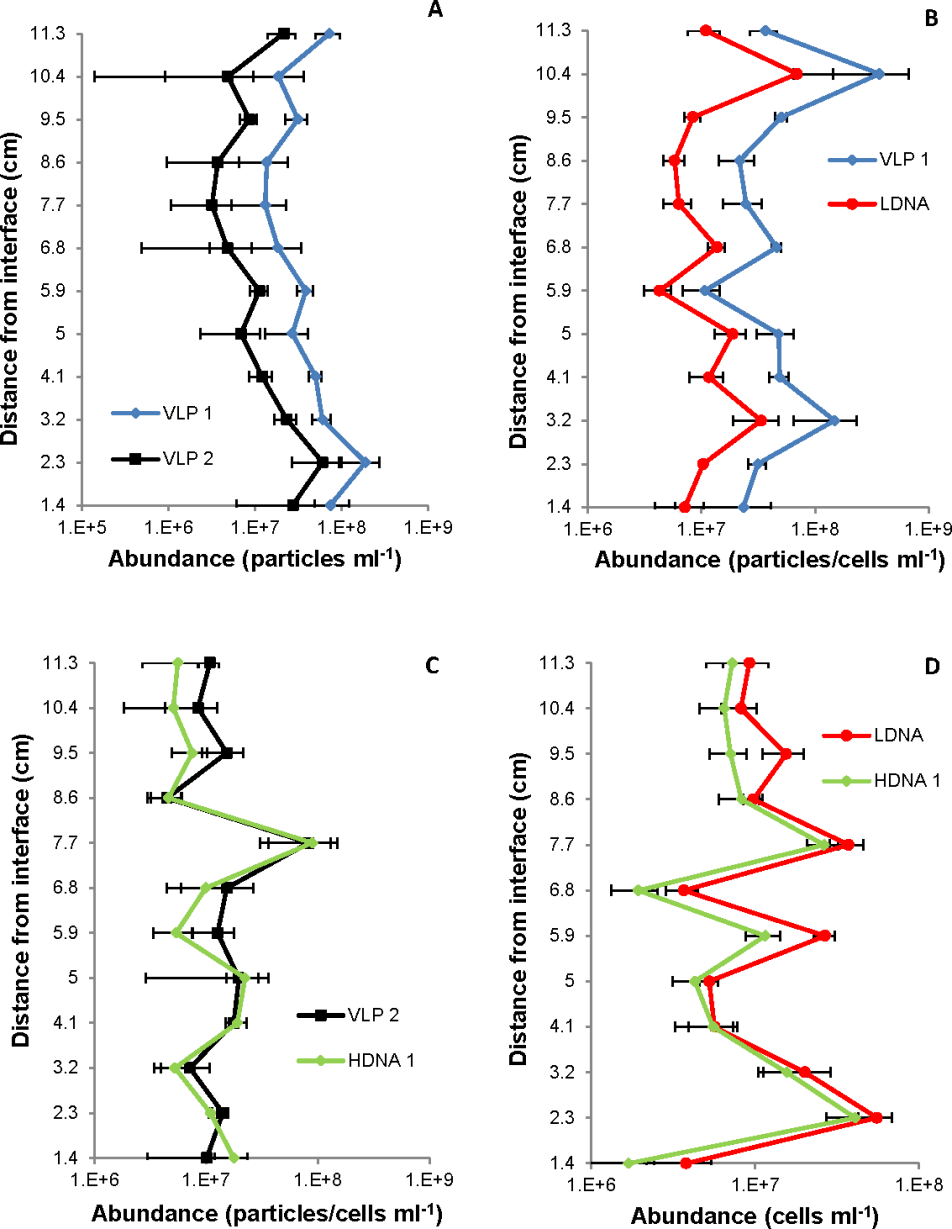
Single vertical profiles showing correlations between VLP and prokaryotic subpopulations. (A) VLP 1 and VLP 2, (B) VLP 1 and LDNA at the sediment-water interface (r = 0.99, p < 0.0001); and (C) VLP 2 and HDNA 1, (D) LDNA and HDNA 1 at the air-water interface (r ≥ 0.97, p < 0.0001). Error bars represent 95% confidence intervals.

To identify potential relationships between plankton populations, Pearson correlation coefficients were run for each subpopulation pair at the air-water interface and sediment-water interface. From the 108 possible single vertical profile subpopulation correlations between VLP 1, VLP 2, LDNA and HDNA, 92 were significantly correlated with an r value ≥ 0.79 (p ≤ 0.003, n = 108) (Fig 5A and 5B). Of these correlated profiles, all of VLP 1 and VLP 2, 16/18 of the VLP 1 and LDNA and VLP 2 and LDNA, 15/18 of the VLP 2 and HDNA, 14/18 of the LDNA and HDNA and 13/18 of the VLP 1 and HDNA subpopulation profiles were correlated (S4 Table).

### Spatial Autocorrelation

#### Moran’s I and Geary’s C

Significant spatial autocorrelation values were seen in 29% of the prokaryotic and VLP subpopulations. Significant Moran’s I values were present in VLP 1 and VLP 2 at 1 out of 3 of the sediment-water interface environments. The range of Moran’s I values for the VLP subpopulations was -0.07 to 0.05 with significance being seen at I values of 0.05. All prokaryotic subpopulations had non-significant Moran’s I values with the range of I values being -0.02 to 0.07 (S5 and S6 Tables).

Significant Geary’s C values were seen in all subpopulations at 1 out of 3 of the sediment-water interface environments, as well as HDNA at 1 out of 3 of the air-water interface environments, whilst their corresponding Moran’s I values were non-significant. The range of Geary’s C values for the prokaryotic subpopulations was 0.97 to 1.16 with significance seen at C values of ≥ 1.13, whilst the range of Geary’s C values for the VLP subpopulations was 0.95 to 1.17 with significance seen at C values of ≥ 1.16 (S5 and S6 Tables).

#### Moran and Geary Correlograms

**Fig 6:**
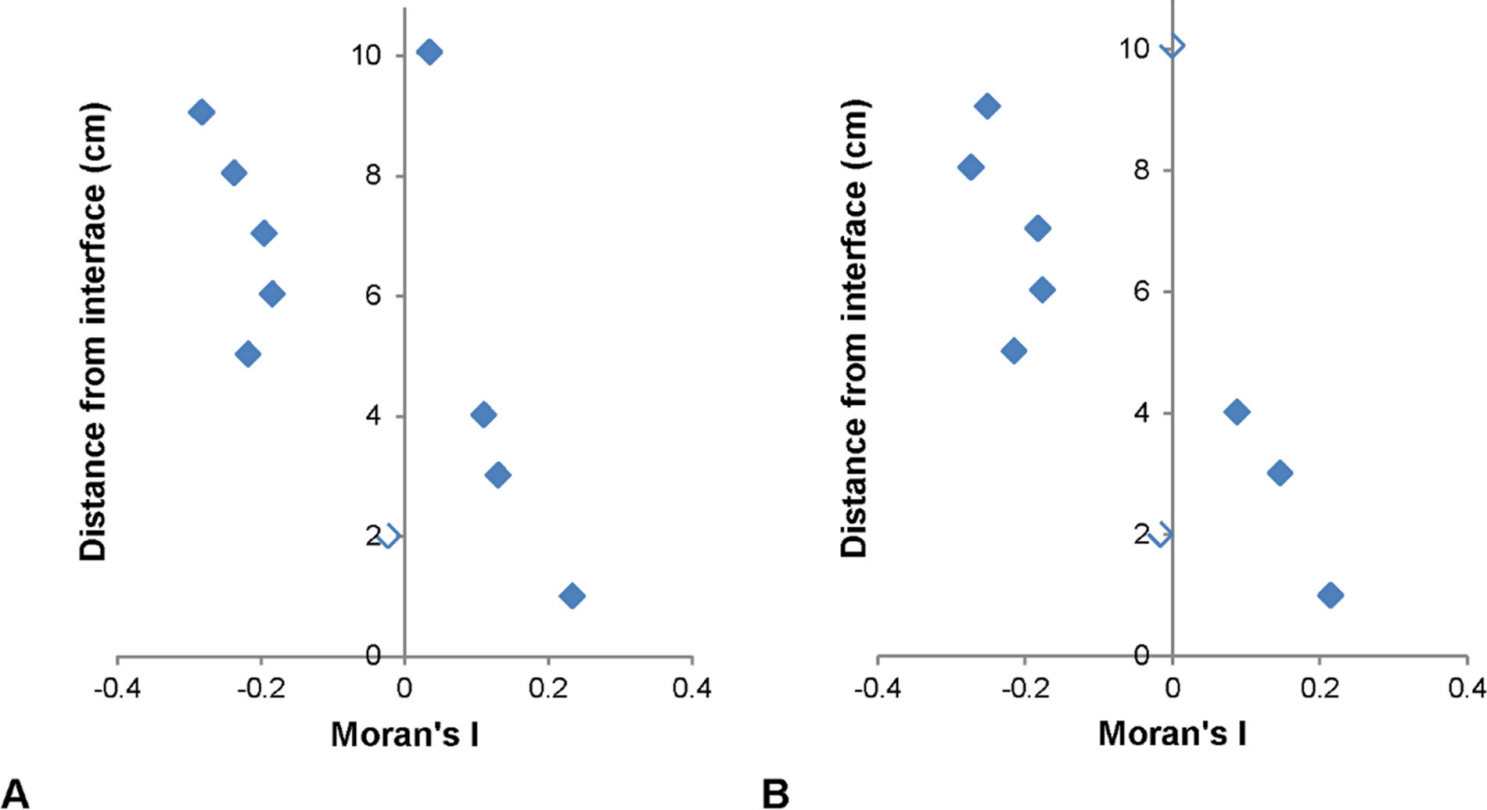
Significant Moran correlograms. (A) VLP 1 and (B) VLP 2 at the sediment-water interface. Unfilled data points indicate non-significance.

**Fig 7:**
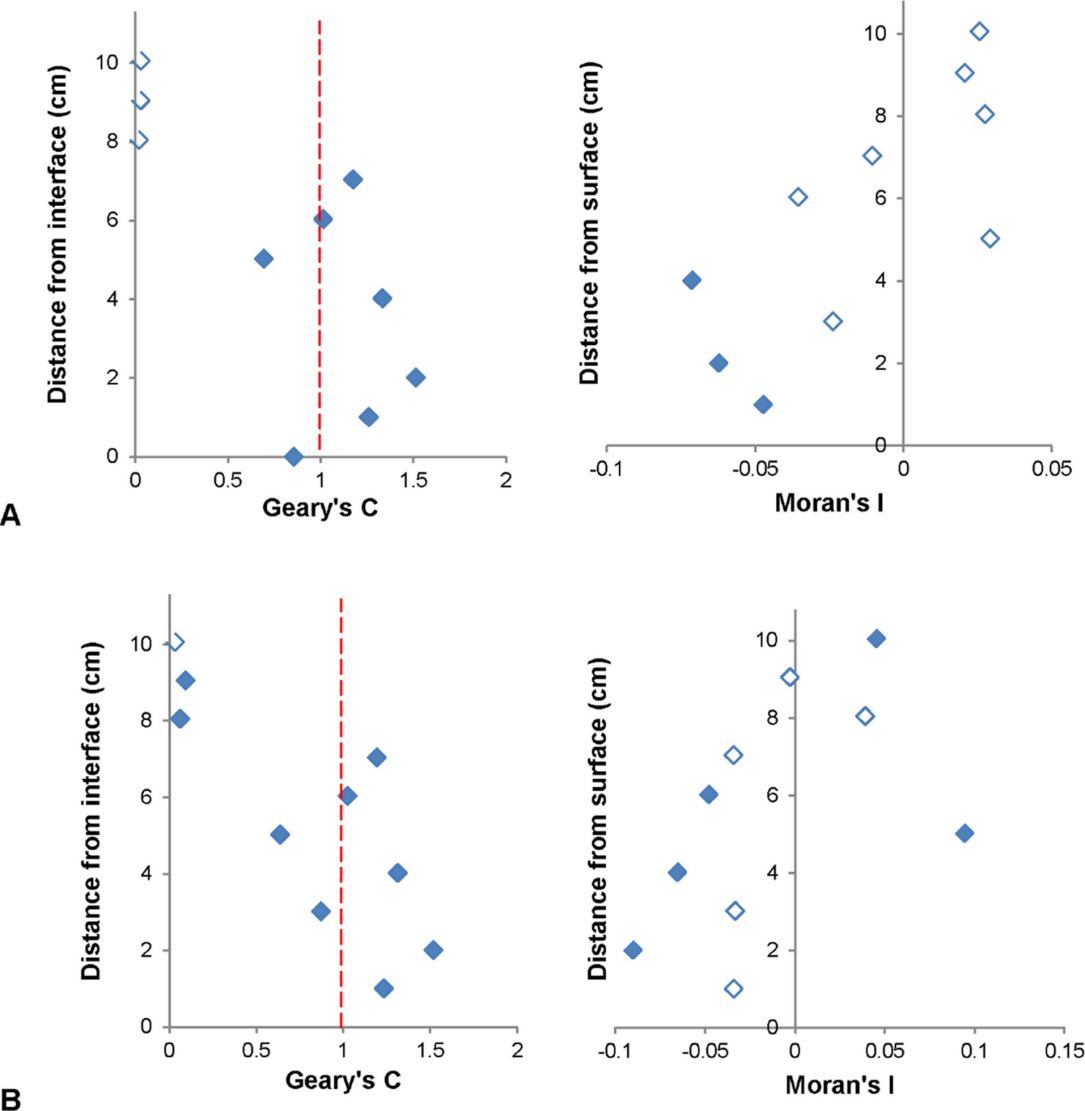
Significant Geary correlograms and corresponding non-significant Moran correlograms. (A) VLP 1 and (B) LDNA at the sediment-water interface. Unfilled data points indicate non-significance. Dashed red lines in Geary correlograms indicate point of spatial independence, with values above indicating negative spatial autocorrelation and values below indicating positive spatial autocorrelation.

Significant Moran correlograms were only found for VLP subpopulations at 1 out of 3 of the sediment-water interface environments (Fig 6A and 6B). Significant Geary correlograms were seen in all subpopulations for 1 out of 3 of the sediment-water interface environments, as well as HDNA at 1 out of 3 air-water interface environments, whilst their corresponding Moran correlograms were non-significant (Fig 7A and 7B). Moran correlograms showed a general trend of positive to negative spatial association whilst Geary correlograms showed alternation between positive and negative spatial autocorrelation with no clear pattern amongst all significant correlograms (Figs 6 and 7).

## Discussion

### Microscale Microbial Patchiness

It is now acknowledged, at least for marine systems, that important microbial processes occur at scales of micrometres to centimetres. Here we tested the hypothesis that microscale microbial communities within freshwater systems exhibit similar patchy abundance distributions as previously observed in marine systems [2, 9, 16, 17, 23, 25, 74, 75]. Our findings showed variations in abundance of 107 and 80.5 fold over 0.9 cm in VLP and prokaryotic subpopulations, indicating heterogeneity consistent with marine systems where it is attributed to mixing and aggregation. However, the magnitude of the gradients was higher than the previously observed maxima of 45 fold variation over 0.9 cm reported for bacterioplankton in marine systems [17]. This potentially indicates that microscale patchiness within interface microenvironments may be higher in freshwater rather than marine systems and that microscale heterogeneity exists at scales that directly impact microbial interactions.

However, as flow and shear patterns are often unique within fluvial systems, the microbial patchiness observed may not be the same or similar between other freshwater systems. For instance, less mixing in lakes compared to flowing waters could potentially result in different levels of microscale patchiness, perhaps due to lake snow or more stable phytoplankton cell distributions. Also, as aggregates in riverine systems are smaller in size than those observed in lakes and marine environments, due to their exposure to contact shear force, this could impact on the level of heterogeneity observed in microbial communities [29–37]. Hydrological and seasonal patterns, such as algal blooms, leaf fall or terrestrial material inflow during flood events, will also impact microbial patchiness due to their observed effects on particle-attached microbial communities [29, 32].

Previously, studies of microbial distributions in freshwater systems often relied on bulk phase, large scale sampling where individual samples were separated by metre to kilometre scales [18–22]. This sampling approach misses the important ecological associations that occur between individual microbial populations at micrometre to centimetre scales. In this study, the vast differences in microbial biomass from one sample point to the next would have been missed had bulk phase sampling been employed, hence influencing estimates in carbon flow. In addition, the results provide further evidence that nutrient exchanges may happen rapidly over very short distances [1, 25]. Here, the ability to collect two-dimensional profiles at a high resolution has allowed discrimination between single point hotspots and adjacent low background values (Fig 3). These single point hotspots and adjacent low background values were responsible for the large fold changes in this study, and show the utility of this two-dimensional sampling technique to provide enhanced scale resolution, as seen previously in marine systems [17]. Our results suggest that where accurate abundance estimates are needed, large scale sampling may improve accuracy by including a few high resolution samples.

The steep adjacent gradients from one sample point to the next in this study illustrate the presence of microscale heterogeneity primarily due to the presence of hotspots. Hotspots in the microscale distributions of prokaryotes and VLPs are well accepted in marine systems, having been found in coral reefs [74], estuaries [16, 17, 23] seawater aquarium [25], eutrophic coastal waters [2, 13, 16, 23, 75] and oligotrophic open ocean systems [16].

The hotspots observed may indicate microbial interactions with suspended particulate matter. For instance, river snow particles can contain high microbial abundances, with these abundances often exceeding the abundance in the water column [33]. These particle associated microbial hotspots can contribute to a significant amount of production and activity [35–38]. Viral attachment to particulate matter can lead to viral abundance hotspots due to prolonged survival or increased phage production and transduction [29, 39–42]. This increased viral production on particles can also result in viral abundance hotspots within the free-living portion of the water column [29].

Nutrient patches may also explain the hotspots in freshwater prokaryote and VLP populations. Nutrient patches are small, being micrometres to centimetres in size and often short-lived, lasting seconds to minutes. These are generated by events such as algal lysis and the sinking of organic particles [25, 26, 48, 51]. As the sampling resolution was larger than the Batchelor scale, chemical gradients could exist from one sample point to the next. These chemical gradients and nutrient patches attract chemotactic bacteria which form clusters around the high nutrient areas [26, 76]. The bacterial accumulation favours viral infection due to high host density allowing viruses to infect multiple bacteria [25, 48]. The abundance patterns that were observed suggested that bacteria had chemotactically responded to nutrient patches, leading to high bacterial abundance at some locations, followed by viral lysis of some of these bacterial species, and hence associated high viral abundance.

### VLP and Prokaryote Subpopulations

The VLP 1 subpopulation was the most abundant which is consistent with previous studies [77, 78] (Fig 1). Previous studies have also shown VLP 1 to contain bacteriophage [58, 79, 80]. However, recent work could not rule out the presence of algal and cyanobacterial viruses in the VLP 1 region [81] and additionally identified active lytic bacteriophage, specifically myoviruses, within VLP 2 FCM signatures [82].

### VLP to Prokaryote Ratio

The mean VPR found in this study was much lower than previous freshwater studies, with the most similar system being a eutrophic subtropical Australian river, which had a minimum VPR of 3.0 [22, 83]. However, this minimum VPR was seen during the summer, which is in contrast to this study which sampled during winter. As higher VPRs are typically seen in productive environments, which are suggested to be due to higher nutrient levels favouring maximum prokaryotic growth and productivity rates, the low VPRs obtained in this study may indicate a less productive system [65, 83–85]. The results confirm the mechanisms for large scale viral and bacterial interactions.

### VLP and Prokaryote Relationships

The vertical profiles of 85% of the prokaryotic and VLP subpopulations were correlated and the VPR values at each sample area were consistent, implying a tight-coupling between prokaryotic and VLP communities (Fig 5). Previous studies have shown positive interdependence between VLP abundance and prokaryotic numbers [86] and subpopulation correlations in marine systems for VLP 1 and LDNA and VLP 2 and HDNA leading to the belief VLP populations are the phage of the prokaryotic populations [3, 15, 25, 87]. The correlations indicate mutual succession of VLPs and prokaryotes, where cell growth is in equilibrium with cell lysis.

The strong correlations may be due to pre-lytic events where bacterial numbers are high and viral numbers are high as was seen in the abundance values [16, 88]. Also, as sampling occurred in winter when nutrient concentrations are minimal and bacterial productivity is at its lowest, lysis would be low and lysogenic viral activity would dominate, causing bacterial and viral numbers to remain relatively constant [78, 89]. Correlations may also represent phytoplankton bloom demise, where bacterial numbers and productivity are high leading to high viral numbers that can last for days to weeks [66, 90–92]. In addition, correlations could indicate nutrient patches attracting bacteria and consequently viruses to high host density areas [76].

Microscale prokaryote and VLP distributions showed different hotspot and coldspot patterns. The two-dimensional contour plots at the air-water interface showed prokaryote hotspots but the absence of VLP hotspots 6 cm from the interface surface (Fig 4). This indicates dynamic differences between prokaryotes and VLPs, perhaps representative of bacterial accumulation around a nutrient source prior to viral attack, or a suspended biofilm particle that is impenetrable to viruses.

### Spatial Autocorrelation within Microbial Subpopulations

Spatial autocorrelation analysis revealed 29% of the prokaryotic and VLP subpopulations were non-randomly distributed at 0.9 cm distances on the local and global scale. Previously significant Moran’s I values have been observed in marine bacterioplankton, however not for VLP subpopulations [17]. As significant Moran’s I values were positive and significant Geary’s C values were > 1, this indicates positive and negative spatial autocorrelation and hence regions of clustering and dispersion, which were present as hotspots and coldspots in two-dimensional abundance distributions [72]. Hotspots may indicate nutrient patches and subsequent bacterial accumulation and viral lysis, whilst coldspots could indicate low nutrient concentration regions where bacterial abundance and productivity is low and lysogeny is favoured [1, 48, 68, 93–95].

Skewing was present in the results, with non-significant Moran’s I values but significant Geary’s C values found for VLP 1, VLP 2, LDNA and HDNA at 1 out of the 3 sediment-water interface environments and HDNA at 1 out of the 3 air-water interface environments (Fig 7). This indicates, although these subpopulations were spatially dependent on the global scale, on the local scale they showed negative spatial autocorrelation areas [69]. This skewing was due to extreme outlier values, i.e. hotspots, which were almost an order of magnitude higher than the rest of the dataset therefore indicates the importance of using Geary’s C in conjunction with Moran’s I.

The significant Moran’s I values in this study and in Dann *et al*. [17] and Waters *et al*. [68] are much lower than the perfect clustering values of +1 indicated by Moran [72]. In Dann *et al*. [17], who looked at microscale virio- and bacterioplankton distributions in marine habitats, significant Moran’s I and Geary’s C values ranged from 0.04 to 0.07 and 0.87 to 1.02 compared to 0.02 to 0.07 and 0.95 to 1.17 in this study. In addition, Waters *et al*. [68], who looked at phytoplankton distributions over 2 cm and 4 cm scales, showed Moran’s I values between 0.08 and 0.18 were indicative of clustering. As previous uses of Moran’s I related to large scale analyses, the lower Moran’s I values obtained in this study could perhaps be characteristic of microscale microbial studies.

## Conclusion

Here we report microscale patchiness in freshwater microbial communities with abundance variations of 107 and 80.5 fold over 0.9 cm for VLP and prokaryotic subpopulations. This indicates that within freshwater ecosystems microbial interactions are likely to differ markedly at the microscale. The pattern of variation is consistent with observations in marine systems for variation caused by mixing and aggregation and therefore suggests, as with marine systems, that bulk phase sampling will not provide accurate representation of the dynamics of microbial processes within freshwater systems.

Spatial autocorrelation analysis showed VLP and prokaryotic subpopulation distributions were non-random and spatially dependent due to heterogeneous hotspots and coldspots. This indicates that the dissipation rates within slow flowing river systems allow nutrient patch formation with lifetimes that exceed bacterial chemotaxis rates.

## Supporting Information

S1 Table Mean abundances of VLP subpopulations. (DOCX)

S2 Table Mean abundances of prokaryotic subpopulations. (DOCX)

S3 Table VLP to prokaryote ratio of the mean abundances. (DOCX)

S4 Table Corresponding r and p values for correlated prokaryotic and VLP subpopulation profiles. (DOCX)

S5 Table Moran’s I and Geary’s C values for prokaryotic subpopulations. (DOCX)

S6 Table Moran’s I and Geary’s C values for VLP subpopulations. (DOCX)
