# Supplementary material for: Distributions of Virus-Like Particles and Prokaryotes within Microenvironments
Source: PLoS One. 2016 Jan 19;11(1):e0146984. doi: 10.1371/journal.pone.0146984 (PMC4718716; doi:10.1371/journal.pone.0146984)
Supplement: S1 Table — (DOCX) [file pone.0146984.s001.docx]

**S1 Table.** Mean VLP abundances at the air- and sediment-water interface. The 95% confidence intervals are included for each mean abundance.

| **Interface** | **Subpopulation** | **Abundance**  10^7^ x cells ml^-1^ (95%CI, n) |
| --- | --- | --- |
| SWI* | VLP 1 | 5.8 (1.2, 108) |
|  | VLP 2 | 1.9 (0.4, 108) |
|  | VLP 1 | 6.7 (1.6, 108) |
|  | VLP 2 | 2.1 (0.5, 108) |
|  | VLP 1 | 5.9 (3.1, 108) |
|  | VLP 2 | 1.7 (0.9, 108) |
| AWI* | VLP 1 | 5.4 (1.0, 108) |
|  | VLP 2 | 1.7 (0.3, 108) |
|  | VLP 1 | 6.1 (1.7, 108) |
|  | VLP 2 | 1.8 (0.6, 108) |
|  | VLP 1 | 5.9 (1.3, 108) |
|  | VLP 2 | 1.8 (0.4, 108) |

*AWI = air-water interface, SWI = sediment-water interface
