# Supplementary material for: Distributions of Virus-Like Particles and Prokaryotes within Microenvironments
Source: PLoS One. 2016 Jan 19;11(1):e0146984. doi: 10.1371/journal.pone.0146984 (PMC4718716; doi:10.1371/journal.pone.0146984)
Supplement: S2 Table — (DOCX) [file pone.0146984.s002.docx]

**S2 Table.** Mean prokaryotic abundances at the air- and sediment-water interface. The 95% confidence intervals are included for each mean abundance. Values indistinguishable from background noise were excluded.

| **Interface** | **Subpopulation** | **Abundance**  10^7^ x cells ml^-1^ (95%CI, n) |
| --- | --- | --- |
| SWI* | LDNA | 1.5 (0.3, 108) |
|  | HDNA 1 | 0.9 (0.2, 108) |
|  | LDNA | 1.6 (0.3, 108) |
|  | HDNA 1 | 1.0 (0.3, 106) |
|  | LDNA | 1.3 (0.5, 108) |
|  | HDNA 1 | 1.0 (0.6, 108) |
| AWI* | LDNA | 1.5 (0.2, 108) |
|  | HDNA 1 | 1.5 (0.4, 108) |
|  | LDNA | 1.4 (0.3, 108) |
|  | HDNA 1 | 1.0 (0.4, 108) |
|  | LDNA | 1.5 (0.2, 108) |
|  | HDNA 1 | 1.2 (0.7, 105) |

*AWI = air-water interface, SWI = sediment-water interface
