# Supplementary material for: Distributions of Virus-Like Particles and Prokaryotes within Microenvironments
Source: PLoS One. 2016 Jan 19;11(1):e0146984. doi: 10.1371/journal.pone.0146984 (PMC4718716; doi:10.1371/journal.pone.0146984)
Supplement: S3 Table — (DOCX) [file pone.0146984.s003.docx]

**S3 Table.** VLP to prokaryote ratio at the air- and sediment-water interface.

| **Interface** | **VPR** |
| --- | --- |
| SWI* | 3.2 |
|  | 3.4 |
|  | 3.3 |
| AWI* | 2.4 |
|  | 3.3 |
|  | 2.9 |

*AWI = air-water interface, SWI = sediment-water interface
