# Supplementary material for: Distributions of Virus-Like Particles and Prokaryotes within Microenvironments
Source: PLoS One. 2016 Jan 19;11(1):e0146984. doi: 10.1371/journal.pone.0146984 (PMC4718716; doi:10.1371/journal.pone.0146984)
Supplement: S4 Table — (DOCX) [file pone.0146984.s004.docx]

**S4 Table.** Corresponding r and p values for correlated prokaryotic and VLP subpopulations profiles.

| **Subpopulation profile** | **R value** | **P value** |
| --- | --- | --- |
| VLP 1 and VLP 2 | ≥ 0.88 | ≤ 0.0002 |
| VLP 1 and LDNA | ≥ 0.85 | ≤ 0.0005 |
| VLP 2 and LDNA | ≥ 0.81 | ≤ 0.0013 |
| VLP 2 and HDNA | ≥ 0.79 | ≤ 0.0024 |
| LDNA and HDNA | ≥ 0.83 | ≤ 0.0008 |
| VLP 1 and HDNA | ≥ 0.79 | ≤ 0.0025 |
