# Supplementary material for: Distributions of Virus-Like Particles and Prokaryotes within Microenvironments
Source: PLoS One. 2016 Jan 19;11(1):e0146984. doi: 10.1371/journal.pone.0146984 (PMC4718716; doi:10.1371/journal.pone.0146984)
Supplement: S5 Table — (DOCX) [file pone.0146984.s005.docx]

**S5 Table.** Moran’s I and Geary’s C values for prokaryotic subpopulations at the air- and sediment-water interface.

| **Interface** | **Subpopulation** | **Moran’s I**  (p-value) | **Geary’s C**  (p-value) |
| --- | --- | --- | --- |
| SWI* | LDNA | 0.02 (n.s) | 0.98 (n.s) |
|  | HDNA 1 | 0.02 (n.s) | 0.97 (n.s) |
|  | LDNA | -0.06 (n.s) | 0.99 (n.s) |
|  | HDNA 1 | -0.06 (n.s) | 0.97 (n.s) |
|  | LDNA | -0.04 (n.s) | 1.16 (0.0001) |
|  | HDNA 1 | -0.04 (n.s) | 1.16 (0.0001) |
| AWI* | LDNA | -0.02 (n.s) | 1.00 (n.s) |
|  | HDNA 1 | -0.04 (n.s) | 0.99 (n.s) |
|  | LDNA | -0.04 (n.s) | 1.06 (n.s) |
|  | HDNA 1 | -0.04 (n.s) | 1.05 (n.s) |
|  | LDNA | -0.07 (n.s) | 1.04 (n.s) |
|  | HDNA 1 | -0.05 (n.s) | 1.13 (0.001) |

*AWI = Air-water interface, SWI = Sediment-water interface.
